# Promoting respectful maternity care in rural Tanzania: nurses’ experiences of the “Health Workers for Change” program

**DOI:** 10.1186/s12913-018-3463-5

**Published:** 2018-08-22

**Authors:** Gail Webber, Bwire Chirangi, Nyamusi Magatti

**Affiliations:** 1 Bruyere Research Institute, 85 Primrose Ave, Ottawa, ON K1R 6M1 Canada; 2 Shirati KMT District Hospital, Rorya, Mara, Shirati, Tanzania

**Keywords:** Maternity care, Health care providers, Disrespect, Abuse

## Abstract

**Background:**

Disrespectful and abusive care of women during their pregnancies has been shown to be a barrier for women accessing health care services for antenatal care and delivery. As part of an implementation research study to improve women’s access to health care services in Rorya District, Mara, Tanzania, we conducted a pilot study training reproductive health care nurses to be more sensitive to women’s needs based on the “Health Workers for Change” curriculum.

**Methods:**

Six series of workshops were held with a total of 60 reproductive health care nurses working at the hospitals, health centres and dispensaries in the district. The participants provided comments on a survey and participated in focus groups at the conclusion of the workshop series. These qualitative data were analyzed for common themes.

**Results:**

The participants appreciated the training and reflected on the poor quality of health care services they were providing, recognizing their attitudes towards their women patients were problematic. They emphasized the need for future training to include more staff and to sustain positive changes. Finally, they made several suggestions for improving women’s experiences in the future.

**Conclusions:**

The qualitative findings demonstrate the success of the workshops in assisting the health care providers to become aware of their negative attitudes towards women. Future research should examine the impact of the workshops both on sustaining attitudinal changes of the providers and on the experiences of pregnant women receiving health care services.

## Background

The global efforts to reduce maternal mortality have led to an emphasis on skilled birth attendance at health care facilities. In understanding why a significant proportion of women continue to avoid facility care, it has become evident that one of the important barriers for women to access maternity care is the disrespectful and abusive attitudes of some health care providers towards women [1]. Our participatory research consultations with community members and policy makers in Rorya District, Mara, Tanzania, confirmed that health care provider attitudes are a key issue to address in order to improve access to care [2]. In our consultations, women described the health care providers using “bad language”, while male partners resorted to offering bribes to the health care providers to ensure better care for their partners.

There is considerable evidence of disrespectful and abusive health care provider attitudes towards women in a number of African settings. In their 2014 study, researchers in Morogoro Region of Tanzania [3] described the spectrum of negative treatment women received at the time of childbirth from their health care providers ranging from feeling neglected or mistreated, to concerns about unpredictable costs, to outright verbal or physical abuse. In their review of Sub-Saharan African health care workers’ attitudes and their impact on sexual and reproductive health care services, Jonas and colleagues [4] examined 35 studies over a 15 year period. They concluded that health care providers’ negative attitudes - in addition to lack of knowledge, limited access to medical equipment and sometimes conservative religious beliefs - limit access of women and particularly adolescents to family planning and antenatal care services.

Other recent African studies have confirmed that women attending health facilities for delivery experience various forms of abuse including verbal, physical and institutionally sanctioned policies which discourage their access to health facilities. In Ethiopia [5], verbal and physical abuse (e.g hitting to encourage women to comply with position changes), and treatment without consent were experienced by women and acknowledged by midwives. Authors of a Kenyan study of 13 institutions [6] found evidence of the following types of abuse towards women during childbirth: physical abuse (including use of force and restraint), verbal abuse, stigma and discrimination based on ethnicity and socio-economic status, failure to meet professional standards of care (including lack of informed consent for exams and procedures, lack of confidentiality and privacy, and neglect and abandonment), lack of rapport with health care providers (including lack of autonomy and detainment), and health system factors. The latter include insufficient resources or staff to service their population and the acceptance of bribery of health care providers to access better care. Qualitative data confirmed that health system challenges limit the ongoing impact of interventions to improve health care provider attitudes [7]. The typology of abuse used in the Kenyan study was developed by Bohren and colleagues [8] in their review of 65 mixed method studies describing abuse during childbirth in 34 countries, including 11 in Sub-Saharan Africa. Their study confirms that while abuse of women in childbirth is context specific, it also appears to be universal to varying degrees.

In preparation for an implementation research project to improve women’s access to health facilities for antenatal care and delivery, we undertook this pilot study to improve health care providers’ attitudes towards pregnant women. This paper reports on the experiences of nurses in a series of workshops based on the “Health Workers for Change” curriculum [9] in Rorya District, Mara Region, Tanzania.

## Methods

The “Health Workers for Change” curriculum includes six workshops covering the following topics titled: “Why I am a health worker”, “How do our clients see us?”, “Women’s status in society”, “Unmet needs”, “Overcoming obstacles at work”, and “Solutions”. The workshops were designed to be highly participatory and include activities in self-reflection such as creating an individual “river of life”, role playing and story telling. The workshops were held over three different days for each group, in six locations within the healthcare institutions (two workshops per day lasting two to 3 hours each), and contained a total of 60 nurses (52 women and eight men). We limited each workshop to 10 participants to encourage active participation of all attendees. The participants all worked at health facilities in Rorya District (hospitals, health centres or dispensaries) and were involved with the care of women and children. The nurses participating in the workshop at a particular location did not necessarily work at that location for we made efforts to bring participants from different health facilities together for the workshops. The workshops were facilitated in Swahili, by two experienced nurses.

After the final workshop, our research team conducted a survey and focus group discussion with each group of nurses. The surveys and focus groups were led by different research team members than the workshop facilitators, in order to allow the participants to express their views of the facilitators in confidence. The survey had an open ended question asking for feedback on the workshops. The purpose of the focus groups were to determine the participants’ experiences in the workshops and seek suggestions for future training and improvement in services. The data from the surveys and focus groups were translated into English and transcribed. N-Vivo software was used for analysis. The transcriptions were coded and analyzed for common themes by the first author. Illustrative quotes were used to demonstrate the themes. All authors reviewed and approved the final manuscript.

## Results

Three themes emerged from the qualitative results from the survey and focus groups: poor quality of health care services, need for future training, and improving women’s experiences.

### Poor quality of health care services

It became clear to the health care providers after attending the workshops, that the quality of health care services they offered was lacking. Multiple participants commented that their attitudes towards their women clients were problematic:

“Women don’t come to health facility because our bad language towards them.” (Focus Group Participant, Kowak Hospital).

“I am very happy about this workshop, because it reminds us about the ethics that we have forgotten and are not following.” (Survey Response, Masonga Health Centre).

“This [workshop] has really changed my behaviour, I thought we were doing the right thing, seriously we were very wrong.” (Focus Group Participant, Kogaja Dispensary).

In addition to their attitudes being a barrier for women attending health facilities, the health care providers also noted the quality of care for women who did attend the health facility was lacking due to insufficient supplies and sometimes limitations of the health care provider’s service:

“Sometimes we have a shortage of supplies so we don’t give satisfactory care.” (Focus Group Participant, Kowak Hospital).

“We also give shallow advice to women and forget some important issues.” (Focus Group Participant, Kowak Hospital).

The workshops provided an opportunity for the health care providers to identify where the weaknesses in quality of service originated. Amongst this group of health care providers serving the rural population of Rorya District, it was clear that their negative attitudes were a major barrier for women, in addition to lack of supplies and appropriate levels of service at the health facility. The health care providers were aware that some women would therefore seek their maternity care elsewhere, particularly with the traditional birth attendants.

### Need for future training

The health care provider participants in the workshops were pleased to have the opportunity to address their negative attitudes, to improve their services. They felt the workshops made a significant impact on their ongoing care, and they could influence others to improve as well. They also requested more time for the seminars in the future.

“I appreciate the seminars. I was able to learn a lot and make changes as needed. I realized the mistakes I was making in giving services to the mothers. I will teach my colleagues so they will also be aware.” (Survey Response, Shirati Hospital).

“I suggest there should be more time for the workshops so that we can learn more about what to tell mothers when they come to health facilities. I would also like the mothers to know that they are learning this, to encourage the mothers who do not want to come.” (Survey Response, Shirati Hospital).

The participants had several suggestions for improving the workshops. In particular, they requested that more of their colleagues have the opportunity to participate in the workshops. For this pilot study, only a fraction of the health care providers working with women and children were chosen to attend, however, for greater impact, they recommended that other health care providers working with pregnant women and children, and those on other services be invited to participate in the workshops.

“The seminar is good, all nurses should participate, not only those working with mothers and children.” (Focus Group Participant, Kowak Hospital).

Some participants also suggested we include management staff, and even women and their partners to widen the impact of the workshops.

“It is good to be with mothers in the seminar so that nurses could hear directly the mother’s opinion.” (Focus Group Participant, Masonga Health Centre).

“I advise that this seminar should have included the employers so they would understand the problems that we face in our work, especially the lack of supplies at the right time. They would see the importance of improving in this area. Also we need to educate the community, especially the men, so they understand the rights of women in society through role play as was demonstrated in the seminar.” (Survey Response, Shirati Hospital).

Many participants insisted that one series of workshops was insufficient to sustain change. They requested that we repeat the workshops for greater impact.

“These workshops should be done again in the future as they give us a chance to improve on giving services to our clients.” (Survey Response, Utegi Health Centre).

“I would like to urge you if it is possible to keep this program going, it is beneficial to us whenever we get this training. It is challenging us to improve services to our clients.” (Survey Response, Rao Hospital).

“I am thankful for the training I got. I realized that I did not know a lot of things that were taught. Now I understand more and I will provide better service to the mothers and children to my ability. I would request more workshops like this in the future.” (Survey Response, Shirati Hospital).

### Improving Women’s experiences

The health care providers participating in the workshops had several suggestions for improving women’s experiences. They emphasized that the continuation of health education about the importance of family planning and safe deliveries was key. Providing financial incentives to community health workers for bringing women for care was also a creative suggestion:

“Establish a system that community health worker can be paid according to the number of pregnant mothers he/she brings or registers.” (Focus Group Participant, Utegi Health Centre).

Another suggestion from several health care providers was to assist women with the cost of transportation to reach the health facility, as this was perceived as a significant barrier for some women. Infrastructure issues such as sufficient, functional equipment, and the provision of health care facilities in convenient locations for women were also mentioned as strategies for improving care. Motivating women to come for care by provision of free baby clothes was proposed by one of the participants. Finally, involving the village leaders to encourage women to attend the health facilities was another suggestion, as the leaders had the benefit of familiarity with the women and would therefore be trusted.

“The village authority should be involved to help mothers to go to health facility because they know them well.” (Focus Group Participant, Shirati Hospital).

## Discussion

The results of the survey comments and focus group discussions support the success of this series of workshops to help health care providers identify their disrespectful attitudes towards their pregnant women patients. There were limitations to this pilot study; only 60 nurses had the chance to participate, and the workshops were covered in three half day sessions. More sessions (one topic per session) with a larger number of more varied participants (including physicians and other providers) would be a goal for future training. Another significant limitation relates to how change is measured: identification of negative attitudes does not equate to an improvement in attitudes. While many of the health care providers acknowledged that their attitudes towards women were inappropriate, it is not clear that the workshops would lead to sustainable improvements in these attitudes. Future research on health care provider attitude change may benefit from the use of validated scales on components such as provider knowledge of clients rights, providers’ emotional health, and working conditions, as was used in the Kenyan study [6]. In addition, it would be useful to investigate patient experiences to determine if health care providers’ intent to improve their attitudes are experienced as improvements in care by pregnant women over time. We plan to explore women’s experiences in the health care system after introduction of the “Health Workers for Change” curriculum in future research through focus group discussions with women after their deliveries.

The participants in this study strongly expressed their appreciation for the workshops in their survey comments and during the focus groups. They acknowledged that they lacked compassion for their women clients and sought to make improvements. They also requested refresher workshops for themselves and their colleagues to help sustain changes in attitudes. These comments suggest that, given appropriate training and support, health care providers in Rorya District are motivated to improve the care of their pregnant patients.

Recently there have been two examples of programs to address disrespectful care in Tanzania described in the literature [10, 11]. In two hospitals in Tanga Region, a before-after design was used to assess a two part intervention [10]. First, a client service charter was produced and revised by multi-level input, including community members. Subsequently, the two hospitals undertook a quality improvement program on their maternity wards addressing disrespectful and abusive treatment at a systems level. The interventions were associated with a 66% reduced odds of women experiencing abuse during childbirth, particularly physical abuse or neglect.

The second Tanzanian example of a program to reduce disrespect was conducted in a large public hospital in Dar es Salaam [11]. Again, two distinct interventions were implemented: an antenatal education program called “Open Birth Days” and a workshop for health care providers based on the “Health Workers for Change” curriculum. The interventions were evaluated using questionnaires of participants, interviews and direct observations of patient-provider interactions at the time of childbirth. Both women and health care providers reported improved communication between providers and patients, health care providers stated they had more empathy for women and better job satisfaction, and women reported increased control and confidence during their deliveries.

The results of these two studies suggest that improvements in care are possible when attitudes of health care providers are addressed, however, changes also need to be made at the systems level to encourage women to access services. As noted in our earlier consultation with community members and policy makers [2], health care provider attitudes are only one of several barriers for women to access prenatal care and delivery. Insufficient supplies and inadequate staffing are two other common concerns, which likely contribute to negative staff attitudes. Working understaffed or without the equipment and medication needed to perform their jobs will make attitude improvements among health care providers unlikely to be sustained. As local governments seek to improve services, they will need to address training, compensation, and retention of appropriate numbers of health care providers to ensure positive work attitudes, in addition to equipping these providers with the necessary tools to practice their skills, including medications to treat their patients.

## Conclusions

Disrespectful and abusive care of maternity patients is a widespread problem with significant implications. In order to improve access of women to health care facilities for their reproductive health care services, the quality of care must improve. Women should not be accessing unskilled providers because they fear the treatment they will receive in a health care facility. The White Ribbon Alliance White for Safe Motherhood and others have also called for promotion of respectful person-centred care in family planning and maternal health as we work towards the Sustainable Development Goals [12]. In recognition that disrespectful maternity care is a barrier for women to access health facilities, the World Health Organization developed a statement “The prevention and elimination of disrespect and abuse during facility-based childbirth” [13] and more recently, a guide to standards for improving maternal and newborn care in the health facility [14]. Criteria such as timely assessment, appropriate evidence-based medical care, effective and sensitive communication, emotional support, and provision of sufficient staff and equipment for service provision are key features of the WHO standards. While meeting all of these criteria will be challenging in resource-limited settings, reducing disrespectful and abusive treatment of women patients will go a long way to improving health care and increasing access to services, thus reducing maternal mortality.

## Abbreviations

WHO: World Health Organization
